# Sequence characterization of *RET* in 117 Chinese Hirschsprung disease families identifies a large burden of de novo and parental mosaic mutations

**DOI:** 10.1186/s13023-019-1194-2

**Published:** 2019-10-30

**Authors:** Qian Jiang, Yang Wang, Qi Li, Zhen Zhang, Ping Xiao, Hui Wang, Na Liu, Jian Wu, Feng Zhang, Aravinda Chakravarti, Wei Cai, Long Li

**Affiliations:** 1 Department of Medical Genetics, Beijing Municipal Key Laboratory of Child Development and Nutriomics, Capital Institute of Pediatrics, Beijing, 100020 China; 2 Department of Pediatric Surgery, Xinhua Hospital, School of Medicine, Shanghai Jiao Tong University, Shanghai Key Laboratory of Pediatric Gastroenterology and Nutrition, Shanghai Institute for Pediatric Research, No. 1665 Kongjiang Rd., Yangpu District, Shanghai, 200092 China; 3 Department of General Surgery, Capital Institute of Pediatrics Affiliated Children’s Hospital, No. 2 Yabao Rd., Chaoyang District, Beijing, 100020 China; 4 Department of Pathology, Capital Institute of Pediatrics Affiliated Children’s Hospital, Beijing, 100020 China; 5 MyGenostics Inc, Beijing, 101318 China; 6 Obstetrics and Gynecology Hospital, Collaborative Innovation Center of Genetics and Development, Fudan University, Shanghai, 200011 China; 7 Center for Human Genetics and Genomics, New York University School of Medicine, New York, NY 10016 USA

**Keywords:** *RET*, Parental mosaicism, High coverage NGS, ddPCR, Chinese HSCR

## Abstract

**Background:**

Hirschsprung disease (HSCR) is an inherited congenital disorder characterized by the absence of enteric ganglia in the distal part of the gut. *RET* is the major causative gene and contains > 80% of all known disease-causing mutations.

**Results:**

To determine the incidence of *RET* pathogenic variants, be they Mendelian inherited, mosaic in parents or true de novo variants (DNVs) in 117 Chinese families, we used high-coverage NGS and droplet digital polymerase chain reaction (ddPCR) to identify 15 (12.8%) unique *RET* coding variants (7 are novel); one was inherited from a heterozygous unaffected mother, 11 were DNVs (73.3%), and 3 full heterozygotes were inherited from parental mosaicism (2 paternal, 1 maternal): two clinically unaffected parents were identified by NGS and confirmed by ddPCR, with mutant allele frequency (13–27%) that was the highest in hair, lowest in urine and similar in blood and saliva. An extremely low-level paternal mosaicism (0.03%) was detected by ddPCR in blood. Six positive-controls were examined to compare the mosaicism detection limit and sensitivity of NGS, amplicon-based deep sequencing and ddPCR.

**Conclusion:**

Our findings expand the clinical and molecular spectrum of *RET* variants in HSCR and reveal a high frequency of *RET* DNVs in the Chinese population.

**Supplementary material:**

**Supplementary information** accompanies this paper at 10.1186/s13023-019-1194-2.

## Introduction

Pathogenic gene variation is a significant contributor to rare diseases, especially in children [1]. Thus, many genetic mutations of early development are inherited by children from their parents through the germline and are present in all cells of that individual, while others, mosaic or somatic mutations, may be acquired postzygotically and are present in only a subset of an individual’s cells [2]. It has long been known that cancer is a mosaic genetic disorder. However, a growing body of research suggests that analogous mosaicism may be a frequent feature in a diverse range of childhood disorders, including cerebral cortical malformations, autism spectrum disorder, epilepsies and other neuropsychiatric diseases [3–6]. In a previous study of Hirschsprung disease (HSCR) families, we identified mosaicism in 6 of 8 (75%) isolated cases [7]. This high frequency was surprising and prompted us to further investigate the frequency and nature of *RET* mosaic pathogenic variants.

HSCR or congenital aganglionosis, a heterogeneous genetic disorder, is characterized by the lack of ganglion cells along varying lengths of the intestine resulting in the major cause of functional obstruction in children. According to the length of aganglionosis, the disorder is categorized into three types: short-segment (aganglionosis segment up to the upper sigmoid colon), long-segment (aganglionosis beyond the splenic flexure) and total colonic aganglionosis (TCA) [8]. The incidence of HSCR varies and is 15, 21 and 28 cases per 100,000 live births in infants with European, African and Asian ancestry, respectively. Genetic studies during the past 25 years have identified rare coding variants in 14 genes that together explain ~ 10% of HSCR cases [9–11]. Of these, the most frequent coding mutations occur in *RET*, which encodes a receptor tyrosine kinase that regulates the proliferation, differentiation and migration of the enteric neural crest cells to enteric neurons. However, family studies of these pathogenic variants demonstrate incomplete penetrance and variable expressivity, the causes of which remain largely unexplained [9, 12].

Numerous studies of *RET* pathogenic variants in HSCR show that they occur in 8.9–16.7% of cases with a contribution from de novo variants (DNVs) which occur in the parental germline [13, 14]. However, family studies of these variants are infrequent so that the distribution of Mendelian inherited versus DNVs is unknown, making risk prediction and genetic counselling of HSCR uncertain. Here, we set out to perform a prospective study of 117 HSCR parent-affected child trios to determine the frequency of *RET* Mendelian inherited, parental mosaic or true DNVs. Furthermore, we explored the mutant allele distribution patterns in multiple somatic tissues and gonadal tissue, and compared the detection accuracy of three commonly used molecular methods.

## Subjects and methods

### Subjects

One hundred and eighteen children diagnosed with isolated HSCR (85/33 male/female, 69/23/26 S-HSCR/L-HSCR/TCA; aged 2–18 months, mean = 16.1 months) from 117 pedigrees were recruited and studied here for the first time, together with their parents and siblings (357 individuals in total). Blood samples were collected from each child, their parents and siblings, and genomic DNA was isolated. Genomic DNA from multiple peripheral tissues, including saliva, urine, hair follicles and sperm, when available, was extracted using the TIANamp Micro DNA Kit (Tiangen Biotech, Beijing, China). Paternity testing was performed on a ProFlex PCR System (Applied Biosystems, USA) using the multiplex STR markers from the AmpFLSTR® Identifiler Plus Amplification Kit (Applied Biosystems, USA).

### Genetic analysis

The coding region of *RET* (RefSeq NM_020975.5) and its annotated functional noncoding elements (putative enhancers, promoters, untranslated regions, exon-intron boundaries ranging from − 50 to + 50 bp, etc.) were enriched from genomic DNA using a GenCap Custom Enrichment Kit (MyGenostics, Beijing, China) [15] as previously described. After sequencing, low-quality reads were filtered out, and adaptor sequences were removed using cutadapt software (http://code.google.com/p/cutadapt/, v1.9.1). Next, we used BWA to align reads to the human reference genome (hg19). After removing duplicates with Picard (v2.2.3), single-nucleotide variants (SNV) and small insertions/deletions (INDEL) were identified using the GATK HaplotypeCaller program (v3.7) and VarScan (v2.3.7). We annotated the identified SNVs and INDELs using ANNOVAR (http://annovar.openbioinformatics.org/en/latest/). Short read alignment and candidate SNP and INDEL validation were performed using IGV. To select putative DNVs, the following criteria were used: 1) minimal 10X coverage in patients and parents; 2) a minimal genotype quality score of 10 for both patients and parents; 3) at least 10% of the reads showing the alternative allele in patients; and 4) not more than 10% of the reads showing the alternative allele in parents. To predict whether a missense change is damaging to the resultant protein function or structure, the following criteria were used: the evolutionary conservation of an amino acid with GERP, the location and context within the protein sequence with InterPro, and the biochemical consequence of the amino acid substitution using SIFT, PolyPhen and MutationTaster.

### Quantification of mosaicism

To validate and quantify putative mosaic events, ~ 12 ng of DNA was used per ddPCR reaction, using previously described methods [16, 17]. Analysis was performed using QuantaSoft software with wells < 8000 total droplets excluded from analysis. Mutant (FAM) and wild-type (HEX) droplet fluorescence were read on the QX200™ Droplet Digital™ PCR System. Alternate allele frequency was calculated as the percentage of mutant-positive droplets divided by the total number of DNA-containing droplets. Multiple wells were merged for analysis, and Poisson confidence intervals were defined using QuantaSoft software (Bio-Rad, Hercules, CA). Samples were deemed “positive” when the 95% Poisson confidence intervals did not overlap the wild-type negative control. Although some samples showed a few positive droplets, they were still deemed negative when their 95% confidence intervals overlapped with wild-type results. Additional statistical analysis was performed in R-Studio (Boston, MA).

## Results

### Novel *RET* coding-region variants detected in 117 families with HSCR

On average, 823.3 million cleaned reads of 100-bp length were generated per sample, except for XHYY019, a male patient with short segment HSCR, which had 74.3 million cleaned reads of 100-bp length. We achieved a minimum of 20-fold coverage per base on average for 99.7% of the target region at a mean coverage of 2962 reads (Basic QC metrics are shown in Additional file 1: Table S1, Table S2). Altogether 16 patients (from 15 families) were discovered to carry *RET* coding-region variants, out of 118 cases (13.6%) but two of these were full siblings. Thus, the variant detection frequency is 15/117 or 12.8%. Note that, two independent probands had the same variant (p. Arg897Gln) and the 14 unique variants consisted of 2 nonsense (p. Arg180*, p. Arg770*), 1 frameshift (p. Val282Valfs*71), 1 splicing (c. 2608-3C > G), 9 missense (p. Ser32Leu, p. Gly93Ser, p. Arg231Cys, p. Gln421Pro, p. Asp489Asn, p. Gly605Asp, p. Gly731Glu, p. Arg897Gln, p. Tyr1062Cys) and 1 synonymous (p. Arg1089Arg) variant. 12 of these changes are absent in both CMDB and gnomAD databases, one (p. Tyr1062Cys) is absent in CMDB and has a very low frequency (1.45 × 10^− 5^) in gnomAD, while the last (p. Asp489Asn) may be common (~ 2% in CMDB and 0.2% in gnomAD). Half of these variants (p. Val282Valfs*71, c. 2608-3C > G, p. Arg231Cys, p. Gln421Pro, p. Gly605Asp, p. Gly731Glu, p. Arg1089Arg) have never been reported in HSCR patients before. Variant annotation suggests that 11 of 14 (78.6%) variants in this sample of HSCR cases are likely pathogenic according to the 2015 ACMG Standards and Guidelines (3 null variants that are absent from controls, 3 previously reported pathogenic de novo missense variants that are absent from controls, 1 de novo missense variant that is absent from controls and affects the amino acid known to be pathogenic, 4 de novo missense variants that are absent from controls and predicted to be deleterious by multiple bioinformatic programs) (Table 1) [18].

**Table 1:**
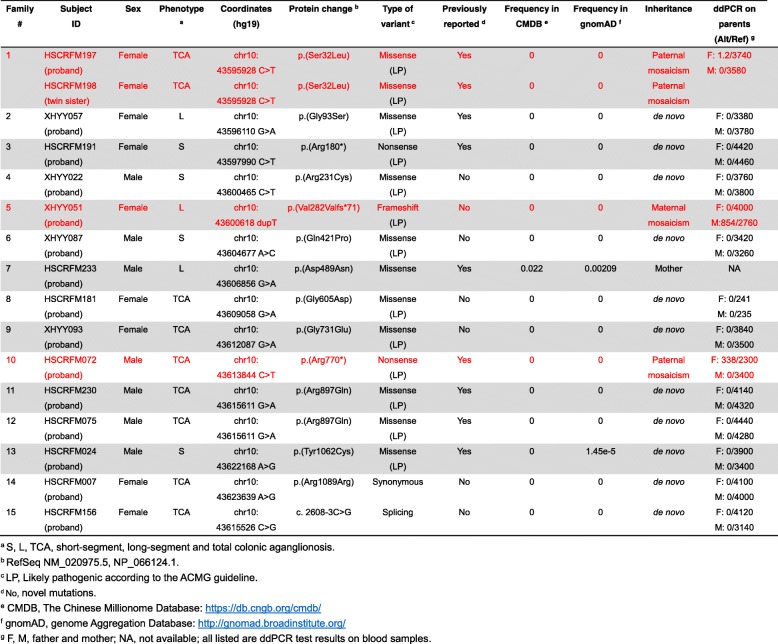
*RET* sequence variants in 15 Chinese HSCR probands with three mosaic events highlighted in red

### Large burden of de novo and parental mosaic pathogenic mutations in HSCR

We next studied the inheritance pattern of each variant using blood DNA of the patients and their parents. One missense variant in family 7 (HSCRFM233) was confirmed to be inherited from the boy’s heterozygous mother. However, surprisingly, the other fourteen families were suspected to have de novo mutations or were parental mosaics. When examined by NGS, the probands showed a mutant: wildtype allelic ratio of 48.0 ± 3.6% (range: 40.5–53.1%) and ddPCR a ratio of 50.0 ± 1.1% (range: 48.4 and 52.1%). The identical twin females in family 1 (HSCRFM197 and HSCRFM198) were first suspected to be post-zygotic mosaics with a mutant allele frequency of 40.5% (alternative allele reads/total coverage: 194/479, similarly hereinafter) and 42.2% (564/1336) according to NGS, but based on ddPCR results of 48.4% (1388/2870) and 50.1% (1836/3666), respectively, were confirmed as true heterozygotes (see Additional file 1: Table S3). The mutant ratios of the other patients are shown as follows, in the order of NGS and ddPCR respectively, with the number of alternative allele reads and total coverage in parenthesis: XHYY057: 46.7% (436/933) vs. 49.4% (1994/4038), HSCRFM191: 48.7% (1110/2281) vs. 52.1% (2000/3840), XHYY022: 50.5% (650/1287) vs. 49.8% (1582/3176), XHYY051: 51.2% (463/905) vs. 49.8% (1824/3660), XHYY087: 50.1% (610/1218) vs. 50.2% (1956/3894), HSCRFM181: 49.7% (441/887) vs. 49.1% (108/220), XHYY093: 50.8% (705/1388) vs. 49.2% (1692/3442), HSCRFM072: 46.7% (307/657) vs. 52.1% (1654/3178), HSCRFM230: 53.1% (129/243) vs. 50.0% (2376/4756), HSCRFM075: 47.0% (379/806) vs. 52.0% (2342/4502), HSCRFM024: 50.4% (1149/2280) vs. 49.6% (1682/3394), HSCRFM007: 48.6% (688/1416) vs. 49.3% (2102/4262), HSCRFM156: 43.4% (162/373) vs. 49.3% (2072/4200).

**Fig. 1:**
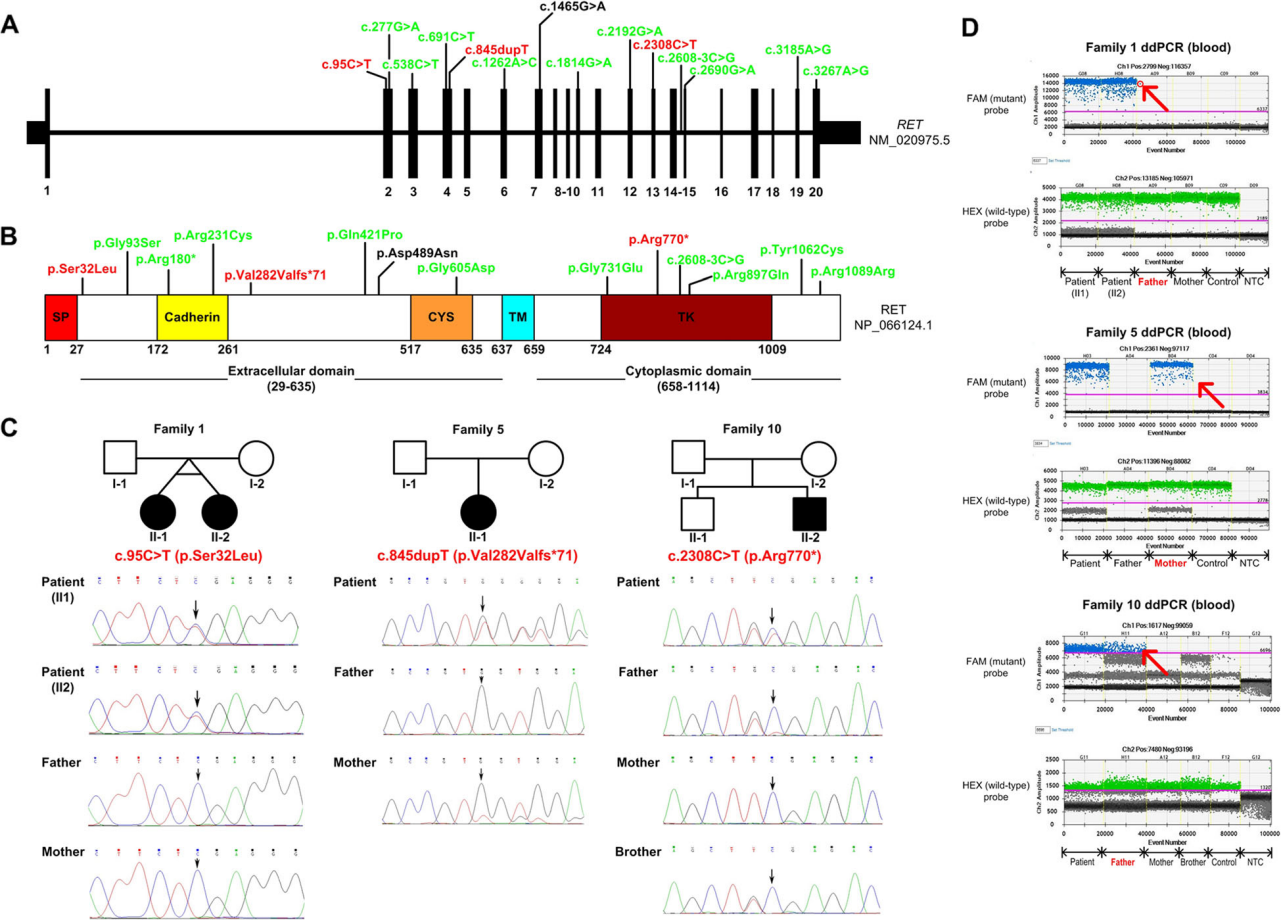
*RET* sequence variants detected in 16 HSCR patients with molecular details on three mosaic variants. **a** Schematic representation of the exon-intron structure of *RET*. Black bars represent exons, and black lines represent introns, with patient mutations indicated above the *RET* genomic structure. **b** Domain structure of RET (GenBank: NP_066124), including the positions (numbers) of identified amino acid alterations. Abbreviations: SP, signal peptide; CYS, cysteine-rich domain; TM, transmembrane domain; TK, tyrosine kinase domain. Inherited, de novo and mosaic variants are shown in black, green and red, respectively, in (**a**) and (**b**). **c** Dideoxy-sequence traces for the three families with *RET* mosaic mutations. In family 1, electropherograms from the patients’ father and mother do not show presence of the variant. In family 5, a small proportion of the mutant c.845dupT allele is present in the proband’s mother, based on both the presence of a small T peak and the reduced relative height of the normal G peak. In family 10, a small proportion of the mutant c.2308C > T allele is present in the proband’s father, based on both the presence of a small T peak and a normal sized C peak. **d** Digital droplet PCR results on families 1, 5 and 10. All positive droplets (those above the threshold intensity indicated by the pink line) are indicated by a red arrow

**Fig. 2:**
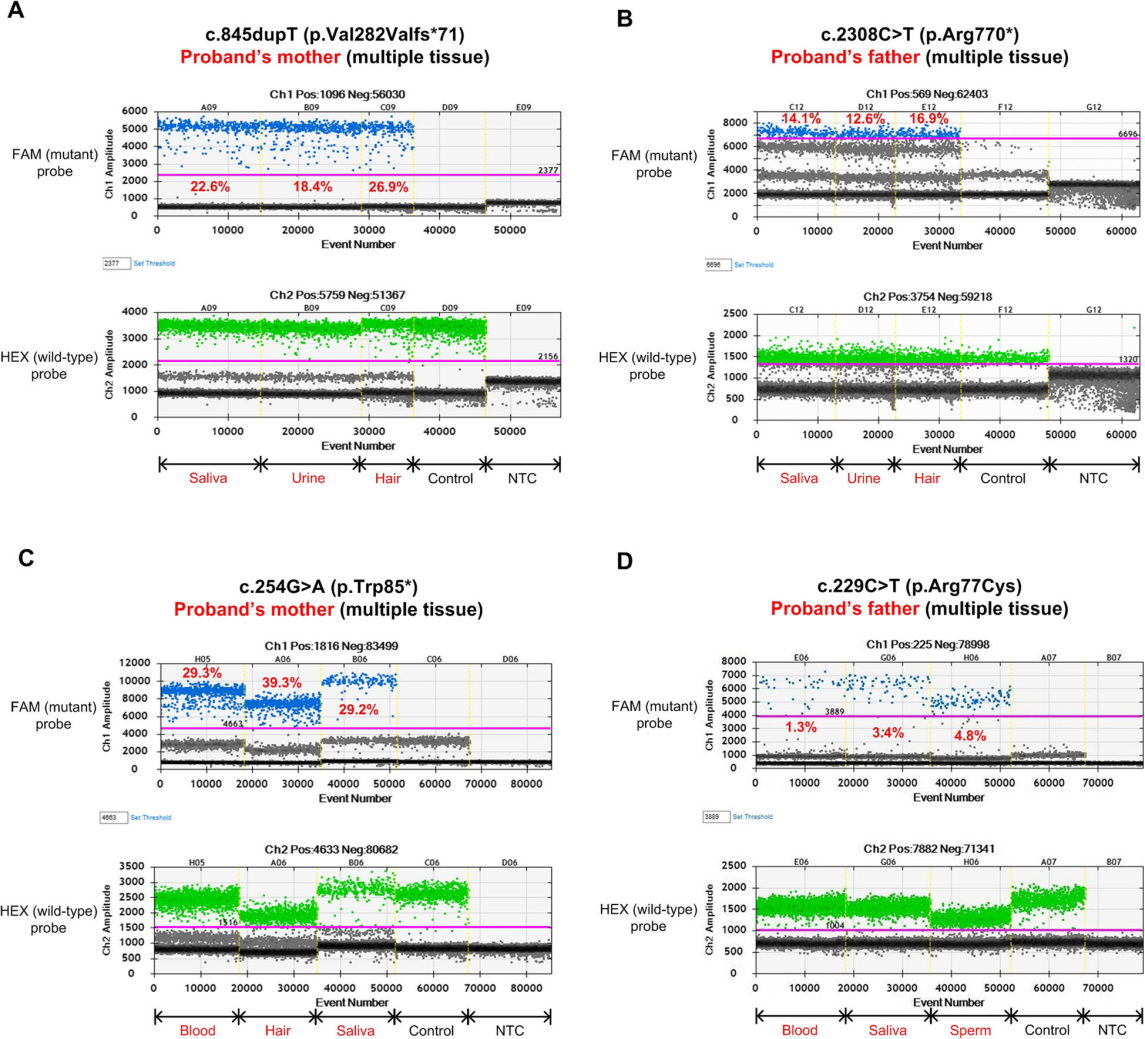
Digital droplet analyses of 4 mosaic variants. Two mosaic variants (**a**, **b**) were discovered in the current study, and two (**c**, **d**) discovered earlier were used as positive-controls. Variant information, alternative-allele frequency and the sample type used are provided for each mutant. Each droplet in a sample is plotted as a graph of fluorescence intensity versus droplet number. All droplets above the threshold intensity indicated by a pink line were scored as ‘positives’ and each assigned a value of 1; ‘negative’ droplets (those below the threshold) were assigned a value of 0. These counts provide a digital signal from which to calculate the starting target DNA concentration by statistical analysis of the numbers of positive and negative droplets in a given sample. NTC, non-template control

Nevertheless, true mosaicism was identified in two clinically unaffected parents by NGS at a sequencing depth of 192X and 703X in families 5 (XHYY051) and 10 (HSCRFM072), respectively. Sanger sequencing detected a small mutant allele peak in the dideoxy-sequence traces for each of them. ddPCR revealed a similar pattern of the mutant allele frequency distribution among multiple tissues: p. Val282Valfs*71 in family 5: 26.9% in hair (mutant-positive droplets/DNA-containing droplets: 728/2708, similarly hereinafter), 18.4% in urine (746/4046), 23.6% in blood (854/3614) and 22.6% in saliva (690/3050); and p. Arg770* in family 10: 16.9% in hair (374/2214), 12.6% in urine (438/3478), 12.8% in blood (338/2638) and 14.1% in saliva (394/2794). An extremely low-level of paternal mosaicism was missed by NGS at a depth of 674X in family 1 but detected by ddPCR with a very low mutant allele frequency in blood at 0.03% (1/3741) (Figs. 1 and 2). Thus, in the 15 HSCR cases, we identified 1 Mendelian inherited, 3 parental germline mosaics (2 paternal, 1 maternal) and 11 DNVs. Functional annotation of these variants shows that 0, 3 and 8, respectively, are likely pathogenic.

### Detection limit and sensitivity of high-coverage NGS, ADS and ddPCR

To determine the detection limit and sensitivity of the three different mutation analysis methods, we examined six positive-control samples, previously demonstrated to carry pathogenic mosaic mutations in *RET*, using amplicon-based deep sequencing (ADS), NGS and ddPCR. Overall, NGS showed a mosaicism detection performance comparable to that of ADS and ddPCR, while ADS displayed a much more reliable detection accuracy and good sensitivity down to a lower limit of ~ 1%: (1) p. Trp85*: 28.0, 41.9 and 28.3% in blood, hair and saliva by ADS; 26.9% in blood by NGS; 29.3, 39.3 and 29.2% in blood, hair and saliva by ddPCR; (2) p. Gln860*: 2.1 and 2.0% in blood and saliva by ADS; and 1.8% in blood by NGS; (3) p. Arg77Cys: 1.3, 2.9 and 4.0% in blood, saliva and sperm by ADS; 0.9% in blood by NGS; 1.3, 3.4 and 4.8% in blood, saliva and sperm by ddPCR (Fig. 2, Table 2).

**Table 2:**
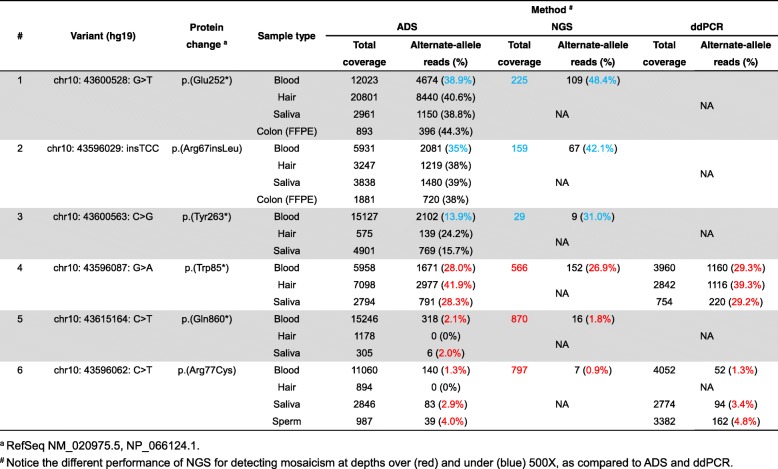
Comparison of amplicon-based deep sequencing (ADS), targeted next-generation sequencing (NGS) and droplet digital polymerase chain reaction (ddPCR) for detection of mosaicism

^a^ RefSeq NM_020975.5, NP_066124.1

^#^ Notice the different performance of NGS for detecting mosaicism at depths over (red) and under (blue) 500X, as compared to ADS and ddPCR

## Discussion

Several lines of evidence suggest that the mechanism of *RET* involvement in HSCR is the result of partial or total loss of RET function, with mutant penetrance depending on the degree of functional loss. We presume that the threshold is > 50% loss because heterozygotes for a *RET* nonsense mutation do not have 100% penetrance in humans [11] but homozygotes for a *Ret* null mutation do have 100% penetrance in mice [19]. One missense variant (p. Asp489Asn) was confirmed to be inherited in a male patient’s unaffected mother in our study. Similarly, multiple putative *RET* mutations were inherited from one of the unaffected parents in a previous study. The underlying mechanism, as stated, is that although a substitution is not thought to be causative of disease in and of itself, it may influence the phenotype, especially given the multigenic nature of HSCR [20, 21]. Here, we identify 2 patients with *RET* nonsense mutations and 1 with frameshift mutation, all resulting in a premature stop codon that is expected to produce non-functional RET. In addition, most of the *RET* HSCR missense mutations involved amino-acids conserved in multiple species and were scattered in the functional domain of RET, which is consistent with the diversity of events predicted to be associated with gene inactivation [21–24]. In brief, those lying within the extracellular domain are proposed to interfere with RET maturation and its translocation to the plasma membrane. Variants residing within the TK domain are likely to reduce the catalytic activity of the receptor, and mutations sitting in the region around Y1062 may compromise the efficiency with which RET binds to its effector molecules. Finally, we also discovered 1 synonymous and 1 splicing variant in families 14 (HSCRFM007) and 15 (HSCRFM156). At face value, these variants are likely benign; however, their absence in large databases suggests that they may have a functional effect acting through activating or abrogating cryptic splice sites or their enhancers [25].

A second intriguing part of this study is the discovery of only one full heterozygote inherited from constitutional heterozygous parent (6.7%) and three heterozygotes inherited from parental mosaics (20%). Genomic mosaicism results from postzygotic events occurring predominantly in early embryogenesis but can arise throughout life and result in genetically distinct cell lines within one individual. Human gastrulation, the process by which the three germ layers are established, is thought to occur at approximately day 16. Primordial germ cells are thought to arise from the primary ectoderm during the second week of development. Therefore, the presence of a somatic variant in blood, saliva (mesodermal tissues), urine (endodermal origin) and hair root bulbs (ectodermal tissue) indicates that the variant arose early enough to potentially also be present in germ cells and is therefore transmissible to the next generation. This high rate of mosaicism suggests that in some families with apparent DNVs, the pathogenic variant is actually mosaic in the parents, and indeed inherited, and that the risk of HSCR in subsequent children is not infinitesimal. This distinction between non-mosaic inherited DNV (heterozygous in proband and variant not detected in parent) and mosaic inherited DNV (heterozygous in proband, and variant detected mosaic in parent) is important for genetic prognosis and counseling. However, it is very difficult to distinguish true DNV from low allele fraction mosaic mutations in reality.

Here, we surveyed 14 families with both NGS and ddPCR on blood DNA. The degree of allelic ratio bias in our NGS results is larger than that in most previous studies, the source of which is still unknown. Among those four where deviation from the expected ~ 50/50 allele ratio of true heterozygosity was observed in NGS, three individuals (HSCRFM197, HSCRFM230 and HSCRFM156) were covered by less than 500X. One exception was HSCRFM198, which had a mutant ratio of 42.2% at a whole coverage of 1336X. In contrast, one sample (HSCRFM181) was covered by less than 500X but ddPCR correctly recognized the mutant status (allele ratio 49.1%), which is not surprising given the nature of the method. NGS can serve as an effective and less expensive technique for screening and quantifying variants; however, it should be noted that many factors may interfere with the results (quality) of the reads/coverage/biallelic ratio by NGS, such as DNA quality (affect baits affinity), biased PCR amplification, sequence context of the variant, pooled DNA isolated from multiple cells as template, the short-read length, sequencing errors and bioinformatic workflow which may filter out biased allele calls. In ddPCR assays, by contrast, template DNA is partitioned into tens of thousands of individual droplets so that at low DNA concentrations the vast majority of droplets contain no more than one copy of template DNA. PCR within each droplet produces a fluorescent readout to indicate the presence or absence of the target of interest, allowing for the accurate “counting” of the number of copies present in a sample [16]. The number of partitions is large enough to assay somatic mosaic events with frequencies down to less than 1%. This excellent accuracy is credited with increased signal-to-noise ratio and removal of PCR bias. As we have shown here, by examining 6 positive-control samples carrying different levels of mosaicism, both ddPCR and ADS surpass the performance of the prevailing NGS and Sanger sequencing.

Interestingly, 11 families (out of 15, 73.3%) were determined to carry non-mosaic inherited DNVs in *RET*, at a significantly higher rate than in any previously reported study: 42.9% in Indonesia, 43.8% in France, and 58.3% in Hong Kong, China [14, 26, 27]. Of these, 72.7% are likely pathogenic. These data raise two issues. First, the pathogenic nature of the DNV needs to be established since *RET* is a commonly mutable gene [28], or rather, its mutants in sperm have a survival advantage [29]. Second, why is the DNV mutation frequency so high? Although our finding may be a chance event it is unlikely because we have observed this before in our studies [7]. A possible and intriguing reason is that many *RET* DNVs may not be disease-causing or be penetrant on their own but can be in a specific RET genetic background that is more permissive in infants with Chinese (Asian) than European ancestry; note that the frequency is also high in the Hong Kong Chinese sample but not the Indonesian one. A candidate for this difference is the *RET* enhancer polymorphism rs2435357 (MCS + 9.7 or RET+ 3) at which a hypomorphic allele that significantly reduces *RET* transcription, has a background allele frequency of 24% (homozygotes ~ 6%) in Europe but 45% (homozygotes ~ 20%) across Asia, a ~ 4-fold difference [30, 31]. MCS + 9.7 does not act on *RET* transcription alone but in concert with at least two other enhancers that also contribute to this genetic background difference [31]. Thus, we hypothesize that this increased widespread susceptibility in China allows a greater number of milder *RET* variants to be HSCR-associated, including DNVs, accounting for the higher frequency of DNVs in Chinese HSCR patients. Regardless, both paternal age and the sequencing sensitivity of different technologies should be taken into account when making the final statement.

Every human gene is subject to random mutation multiple times within each individual. However, most variants are either benign or never reach a fraction high enough to cause disease. Thus, whether a pathogenic variant is disease penetrant or not depends on the physiological function of the encoded molecule and the fraction of cells possessing the mutation in a given tissue. Somatic mutations that lead to a gain of function or growth advantage might cause disease if they are present in even one cell, as in cancer. On the other hand, somatic mutations that lead to a loss of function might need to occur in a larger clonal fraction in order to cause a clinical phenotype. Therefore, for every deleterious somatic mutation there likely exists a threshold mosaic fraction above which the mutation causes disease but below which it does not and so remains undetected [32]. Of course, for de novo changes the penetrance is likely dependent on the number of cells affected, as well as the specific mutation, the disease involved, and the genetic background of the individual. Thus, distinguishing non-mosaic inherited DNV (germline DNV) from true postzygotic DNVs is important, as is the threshold mosaic fraction. These analyses need to be quantitative because in some cases, in clinically significant cortical malformations, the disorder can result from somatic mutations in as few as 1% of cells [33]. The threshold mosaic fraction for HSCR is important to investigate because it is likely a critical determinant of HSCR penetrance and expressivity.

## Conclusion

Together with previously reported cases, our study broadened the clinical and molecular spectrum of HSCR and revealed a large burden of de novo and parental mosaic pathogenic mutations in *RET* in the Chinese population. All the observations indicated that distinguishing non-mosaic inherited DNV from mosaic inherited DNV is important for both genetic prognosis and accurate counseling.

## Supplementary information

**Additional file 1: Table S1.** Summary of the quality of targeted next-generation sequencing data on *RET.* **Table S2.** Quality of targeted next-generation sequencing data on *RET* of all the samples in the current study. **Table S3.** Comparison of mutant ratio in 15 HSCR patients carrying 13 different kinds of *RET* variants examined by targeted next-generation sequencing (NGS) and droplet digital polymerase chain reaction (ddPCR). (DOCX 91 kb)

## Data Availability

All data generated or analysed during this study are included in this published article and its supplementary information files.
