# Supplementary material for: Sequence characterization of RET in 117 Chinese Hirschsprung disease families identifies a large burden of de novo and parental mosaic mutations
Source: Orphanet J Rare Dis. 2019 Oct 30;14:237. doi: 10.1186/s13023-019-1194-2 (PMC6822467; doi:10.1186/s13023-019-1194-2)
Supplement: Supplementary file 1 — Additional file 1: Table S1. Summary of the quality of targeted next-generation sequencing data on RET. Table S2. Quality of targeted next-generation sequencing data on RET of all the samples in the current study. Table S3. Comparison of mutant ratio in 15 HSCR patients carrying 13 different kinds of RET variants examined by targeted next-generation sequencing (NGS) and droplet digital polymerase chain reaction (ddPCR). (DOCX 91 kb) [file 13023_2019_1194_MOESM1_ESM.docx]

**Supplementary Material**

Sequence characterization of *RET* in 117 Chinese Hirschsprung disease families identifies a large burden of de novo and parental mosaic mutations

Qian Jiang, Yang Wang, Qi Li, Zhen Zhang, Ping Xiao, Hui Wang, Na Liu, Jian Wu, Feng Zhang, Aravinda Chakravarti, Wei Cai, Long Li

Correspondence to: Dr. Wei Cai at the Department of Pediatric Surgery, Xinhua Hospital, School of Medicine, Shanghai Jiao Tong University; Shanghai Key Laboratory of Pediatric Gastroenterology and Nutrition; Shanghai Institute for Pediatric Research, No. 1665 Kongjiang Rd., Yangpu District, Shanghai 200092, China (caiw204@sjtu.edu.cn); and Dr. Long Li at the Department of General Surgery, Capital Institute of Pediatrics Affiliated Children's Hospital, No. 2 Yabao Rd., Chaoyang District, Beijing 100020, China ([lilong23@126.com](mailto:lilong23@126.com))

**Table S1. Summary of the quality of targeted next-generation sequencing data on *RET.***

| **Class** | **Raw data (Mb)** | **Cleaned data (Mb)** | **Mapped data (Mb)** | **GC (%)** | **Q20 (%)** | **Depth** | **Fraction of target covered ≥20X** |
| --- | --- | --- | --- | --- | --- | --- | --- |
| Minimum | 89.9 | 74.3 | 73.8 | 39.7 | 81.2 | 304.0 | 96.1 |
| Maximum | 2406.5 | 2117.2 | 2111.6 | 49.7 | 96.6 | 7336.1 | 100.0 |
| Average | 937.4 | 823.3 | 820.4 | 45.2 | 94.2 | 2962.5 | 99.7 |
| Median | 861.3 | 766.2 | 764.8 | 45.4 | 94.6 | 2918.3 | 100.0 |

**Table S2. Quality of targeted next-generation sequencing data on *RET* of all the samples in the current study.**

| **Sample ID** | **Raw data (Mb)** | **Cleaned data (Mb)** | **Mapped data (Mb)** | **GC (%)** | **Q20 (%)** | **Depth** | **Fraction of target covered**  **≥20X** |
| --- | --- | --- | --- | --- | --- | --- | --- |
| 17R005271 | 831.39 | 715.19 | 709.86 | 44.89 | 91.5 | 2481.47 | 100 |
| 17R005272 | 703.3 | 619.66 | 617.56 | 46.12 | 93.88 | 3090.33 | 100 |
| 17R005273 | 973.11 | 859.34 | 856.06 | 45.21 | 94.47 | 4201.51 | 100 |
| 17R005274 | 885.75 | 779.87 | 778.45 | 45.09 | 96.52 | 3488.86 | 100 |
| 17R005275 | 1957.99 | 1735.81 | 1731.85 | 45.51 | 95.42 | 6952.73 | 100 |
| 17R005276 | 1703.93 | 1510.3 | 1507.53 | 45.61 | 95.01 | 5764.15 | 100 |
| 17R005277 | 616.6 | 545.95 | 543.89 | 46.1 | 93.63 | 2573.21 | 100 |
| 17R005278 | 731.66 | 639.02 | 636.37 | 46.78 | 93.78 | 3203.71 | 100 |
| 17R005279 | 544.79 | 474.79 | 471.97 | 43.84 | 92.5 | 1610.87 | 99.56 |
| 17R005280 | 904.74 | 779.47 | 774.82 | 45.06 | 91.97 | 2796.31 | 100 |
| 17R005281 | 1161.79 | 1038.48 | 1035.73 | 45.39 | 94.94 | 4304.5 | 100 |
| 17R005282 | 930.15 | 830.61 | 828.29 | 45.67 | 94.61 | 3605.28 | 100 |
| 17R005283 | 1206.75 | 1073.75 | 1071.46 | 45.28 | 95.59 | 3972.68 | 100 |
| 17R005284 | 509.03 | 436.26 | 434.76 | 46.36 | 93.62 | 2162.03 | 100 |
| 17R005285 | 921.04 | 819.54 | 815.73 | 45.08 | 93.4 | 3563 | 100 |
| 17R005288 | 1022.21 | 896.4 | 894.65 | 45.31 | 96.21 | 4245.91 | 100 |
| 17R005289 | 1056.97 | 926.53 | 924.21 | 45.92 | 95.37 | 3903.11 | 100 |
| 17R005290 | 597.04 | 525.8 | 523.85 | 46.69 | 93.49 | 2512.63 | 100 |
| 17R005291 | 810.2 | 708.97 | 705.88 | 45.83 | 93.54 | 3532.43 | 100 |
| 17R005292 | 1245.39 | 1108.45 | 1102.91 | 45.19 | 93.24 | 4272.26 | 100 |
| 17R005293 | 583.02 | 514.85 | 513.11 | 45.85 | 93.66 | 2389.91 | 100 |
| 17R005294 | 176.62 | 146.68 | 145.66 | 46.94 | 92.62 | 724.45 | 99.96 |
| 17R005295 | 933.64 | 820.36 | 817.64 | 44.27 | 94.16 | 3104.48 | 99.91 |
| 17R005296 | 829.66 | 739.17 | 736.12 | 44.89 | 93.62 | 2633.23 | 100 |
| 17R005297 | 2406.48 | 2117.19 | 2111.6 | 45.18 | 94.64 | 7256.75 | 100 |
| 17R005298 | 1305.83 | 1140.07 | 1137.58 | 45.48 | 95.39 | 4561.33 | 100 |
| 17R005299 | 1152.35 | 1011.72 | 1009.49 | 47.32 | 95.12 | 3354.62 | 100 |
| 17R005300 | 1260.25 | 1125.95 | 1122.89 | 47.22 | 94.92 | 3692.5 | 100 |
| 17R005301 | 1475.42 | 1308.8 | 1305.89 | 46.65 | 95.47 | 3989.9 | 100 |
| 17R005302 | 1530.64 | 1364.91 | 1361.78 | 46.43 | 95.46 | 4263.28 | 100 |
| 17R005303 | 1258.78 | 1119.63 | 1116.64 | 47.05 | 95.04 | 3911.4 | 100 |
| 17R005304 | 1218.13 | 1069.95 | 1067.74 | 46.14 | 95.31 | 3761.24 | 100 |
| 17R005305 | 1315.4 | 1182.99 | 1180.08 | 46.09 | 94.78 | 5039.13 | 100 |
| 17R005306 | 680.4 | 606.2 | 604.88 | 46.64 | 94.98 | 3766.36 | 100 |
| 17R005307 | 1517.59 | 1341.83 | 1338.89 | 44.93 | 95.46 | 4345.78 | 100 |
| 17R005308 | 1366.25 | 1213.28 | 1210.58 | 47.52 | 95.33 | 4304.66 | 100 |
| 17R005309 | 1366.56 | 1196.19 | 1193.73 | 46.06 | 95.52 | 3924.67 | 100 |
| 17R005310 | 1624.83 | 1416.57 | 1413.56 | 45.91 | 95.43 | 6404.35 | 100 |
| 17R005311 | 501.96 | 444.28 | 443.31 | 47.08 | 93.37 | 2380.59 | 100 |
| 17R005312 | 1556.21 | 1349.84 | 1344.48 | 46.74 | 94.43 | 5592.12 | 100 |
| 17R005313 | 1061.34 | 933.68 | 931.66 | 47.8 | 95.37 | 3254.31 | 100 |
| 17R005314 | 1141.41 | 984.74 | 982.5 | 46.44 | 95.45 | 3237.29 | 100 |
| 17R005315 | 1337.41 | 1190.94 | 1188.32 | 45.92 | 95.42 | 4240.84 | 100 |
| 17R005316 | 1556.67 | 1367.51 | 1364.29 | 44.84 | 95.37 | 4742.28 | 100 |
| 17R005317 | 1550.55 | 1381.57 | 1378 | 46.04 | 95.34 | 4336.57 | 100 |
| 17R005318 | 1232.59 | 1096.12 | 1093.89 | 47.14 | 95.54 | 3714.62 | 100 |
| 17R005319 | 1375.54 | 1190.21 | 1187.27 | 47.83 | 94.99 | 4188.65 | 100 |
| 17R005320 | 1999.31 | 1727.85 | 1719.04 | 44.37 | 94.74 | 4130.03 | 100 |
| 17R005321 | 1358.63 | 1203.57 | 1200.58 | 45.99 | 95.27 | 3870.45 | 100 |
| 17R005322 | 1287.97 | 1127.84 | 1125.47 | 45.47 | 95.59 | 3570.34 | 100 |
| 17R005323 | 1564.94 | 1363.23 | 1359.53 | 45.43 | 95.04 | 4223.71 | 100 |
| 17R005324 | 470.48 | 418.35 | 417.05 | 46.1 | 92.81 | 2103.17 | 100 |
| 17R005325 | 1035.84 | 897.87 | 895.29 | 45.17 | 94.44 | 1015.89 | 99.52 |
| 17R005326 | 802.7 | 709.97 | 708.54 | 46.34 | 95.3 | 2769.99 | 100 |
| 17R005327 | 1300.72 | 1138.42 | 1135.94 | 45.69 | 95.6 | 4020.7 | 100 |
| 17R005328 | 1079.07 | 963.65 | 961.34 | 46.69 | 95.34 | 3424.25 | 100 |
| 17R005329 | 1474.61 | 1300.57 | 1297.93 | 44.33 | 95.74 | 3900.26 | 100 |
| 17R005330 | 1670.13 | 1463.56 | 1460.49 | 46.87 | 95.28 | 6793.55 | 100 |
| 17R005331 | 587.9 | 524.31 | 523.29 | 46.85 | 95.64 | 3319.08 | 100 |
| 17R005332 | 751.47 | 652.29 | 649.92 | 46.56 | 94.13 | 2679.58 | 100 |
| 17R005333 | 352 | 310.46 | 309.52 | 46.57 | 92.37 | 1625.67 | 100 |
| 17R005334 | 870.98 | 771.79 | 770.01 | 47.05 | 94.43 | 3648.57 | 100 |
| 17R005335 | 425.38 | 352.33 | 350.66 | 46.98 | 94.2 | 1716.62 | 100 |
| 17R005336 | 1003.07 | 902.84 | 900.38 | 46.36 | 94.73 | 3696.9 | 100 |
| 17R005337 | 1441.19 | 1294.25 | 1290.8 | 46.18 | 95.37 | 4326.81 | 100 |
| 17R005338 | 1133.28 | 1009.3 | 1007.21 | 46.56 | 95.71 | 3500.33 | 100 |
| 17R005339 | 1414.09 | 1257.81 | 1253.69 | 45.9 | 95.01 | 3697.28 | 100 |
| 17R005340 | 1544.39 | 1340.14 | 1336.87 | 47.18 | 95.15 | 4486.48 | 100 |
| 17R005341 | 1305.95 | 1156.98 | 1154.45 | 46.75 | 95.43 | 3812.08 | 100 |
| 17R005342 | 1542.7 | 1376.29 | 1373.15 | 46.19 | 95.52 | 4310.44 | 100 |
| 17R005343 | 1205.51 | 1079.6 | 1077.53 | 47.81 | 95.54 | 3727.24 | 100 |
| 17R005344 | 291.63 | 261.34 | 260.5 | 47.9 | 94.77 | 1063.14 | 100 |
| 17R005345 | 1252.64 | 1117.51 | 1114.97 | 46.18 | 95.32 | 3810.74 | 100 |
| 17R005346 | 1385.91 | 1238.66 | 1235.69 | 47.48 | 95.17 | 4027.64 | 100 |
| 17R005347 | 1294.95 | 1160.21 | 1157.24 | 45.73 | 95.21 | 3850.09 | 100 |
| 17R005348 | 1002.91 | 901.28 | 897.02 | 46.26 | 93.84 | 3535.74 | 100 |
| 17R005349 | 819.92 | 719.99 | 716.45 | 45.25 | 92.91 | 2352.31 | 100 |
| 17R005350 | 784.7 | 697.21 | 695.35 | 46.11 | 94.76 | 3213.57 | 100 |
| 17R005351 | 983.25 | 871.53 | 869.8 | 47.13 | 95.36 | 3195.09 | 100 |
| 17R005352 | 1046.83 | 935.06 | 932.95 | 47.48 | 95.08 | 3676.8 | 100 |
| 17R005353 | 1157.82 | 1027.76 | 1025.41 | 45.85 | 95.28 | 3589.93 | 100 |
| 17R005354 | 1729.45 | 1278.67 | 1275.73 | 46.78 | 95.33 | 3939.47 | 100 |
| 17R005355 | 1685.46 | 1501.78 | 1497.99 | 44.79 | 95.36 | 4387.94 | 100 |
| 17R005356 | 902.3 | 789.57 | 787.95 | 47.23 | 95.14 | 2882.41 | 100 |
| 17R005357 | 1621.32 | 1441.45 | 1438.19 | 46.17 | 95.38 | 4611.17 | 100 |
| 17R005358 | 1690.38 | 1516.5 | 1510.34 | 44.7 | 95.04 | 3750.24 | 100 |
| 17R005359 | 1299.21 | 1170.28 | 1167.64 | 45.87 | 95.34 | 4133.78 | 100 |
| 17R005360 | 1877.96 | 1672.93 | 1669.71 | 44.7 | 95.78 | 6621.9 | 100 |
| 17R005361 | 1374.78 | 1211 | 1207.47 | 44.7 | 94.34 | 4489.54 | 100 |
| 17R005362 | 1612.75 | 1411.25 | 1408.63 | 46.38 | 95.46 | 7058.65 | 100 |
| 17R005363 | 731.4 | 649.29 | 645.97 | 45.3 | 93.5 | 2555.16 | 100 |
| 17R005364 | 599.92 | 521.41 | 519.8 | 44.18 | 94.5 | 2545.67 | 100 |
| 17R005365 | 608.36 | 510.8 | 506.57 | 46.07 | 91.08 | 2098.67 | 100 |
| 17R005366 | 1159.2 | 996.02 | 993.95 | 44.59 | 95.13 | 1080.72 | 98.5 |
| 17R005367 | 1125.02 | 982.21 | 980.41 | 45.23 | 95.3 | 2720.63 | 100 |
| 17R005368 | 576.4 | 516.94 | 516.17 | 47.48 | 94.68 | 3098.42 | 100 |
| 17R005369 | 1404.24 | 1202.37 | 1200.84 | 43.47 | 95.99 | 1321.92 | 97.3 |
| 17R005370 | 741.77 | 625.98 | 623.19 | 44.7 | 93.18 | 617.81 | 98.54 |
| 17R005371 | 1449.73 | 1273.62 | 1271.64 | 45.44 | 95.33 | 4660.44 | 100 |
| 17R005372 | 946.99 | 848.84 | 847.17 | 46.79 | 94.67 | 3815.91 | 100 |
| 17R005373 | 538.94 | 486.84 | 485.77 | 47.77 | 94.31 | 3072.1 | 100 |
| 17R005374 | 687.9 | 617.26 | 615.84 | 46.56 | 94.34 | 3496.4 | 100 |
| 17R005375 | 996.88 | 877.04 | 875.42 | 45.95 | 95.48 | 3518.54 | 100 |
| 17R005376 | 1138.78 | 1006.19 | 1003.91 | 46.57 | 95.04 | 4403.95 | 100 |
| 17R005377 | 1040.43 | 913.83 | 912.41 | 44.61 | 96.16 | 3803 | 100 |
| 17R005378 | 1712.99 | 1501.6 | 1496.87 | 43.82 | 94.02 | 2747.53 | 100 |
| 17R005379 | 141.83 | 120.7 | 120.05 | 48.7 | 93.83 | 683 | 100 |
| 17R005380 | 653.68 | 593.5 | 592.09 | 46.51 | 94.41 | 3280.78 | 100 |
| 17R005381 | 1194.55 | 1040.44 | 1038.72 | 44.26 | 95.77 | 1298.85 | 99.78 |
| 17R005382 | 1536.36 | 1363.57 | 1360.56 | 44.85 | 95.7 | 5521.35 | 100 |
| 17R005383 | 711.58 | 637.9 | 636.65 | 46.09 | 95.37 | 3989.76 | 100 |
| 17R005384 | 1059.71 | 921.27 | 915.82 | 45.3 | 93.1 | 3332.7 | 100 |
| 17R005385 | 1031.4 | 920.16 | 918.08 | 45.72 | 95.33 | 3734.63 | 100 |
| 17R005386 | 509.65 | 455.18 | 454.05 | 46.52 | 92.79 | 2271.73 | 100 |
| 17R005387 | 769.43 | 675.64 | 673.71 | 44.61 | 94.71 | 2348.39 | 100 |
| 17R005388 | 1766.79 | 1571.39 | 1566.82 | 45.57 | 94.91 | 6451.01 | 100 |
| 17R005389 | 890.05 | 766.24 | 764.81 | 45.83 | 95.71 | 3748.1 | 100 |
| 17R005390 | 1324.13 | 1155.09 | 1152.38 | 44.27 | 93.88 | 3439.05 | 100 |
| 17R005391 | 1859.1 | 1627.38 | 1622.59 | 45.31 | 95.35 | 4429.78 | 100 |
| 17R005392 | 1557.57 | 1384.09 | 1381.04 | 44.8 | 94.48 | 4491.96 | 100 |
| 17R005393 | 768.36 | 691.81 | 690.36 | 46.77 | 94.19 | 3790.37 | 100 |
| 17R005394 | 2127.24 | 1870.67 | 1865.45 | 44.48 | 95.18 | 6925.32 | 100 |
| 17R005395 | 957.09 | 834.68 | 831.71 | 44.09 | 94.49 | 1798.73 | 99.2 |
| 17R005396 | 926.59 | 784.96 | 783.68 | 44.96 | 95.36 | 1190.34 | 99.6 |
| 17R005397 | 491.22 | 442.75 | 441.22 | 48.81 | 92.52 | 2778.46 | 100 |
| 17R005398 | 536.36 | 468.96 | 467.96 | 46.93 | 94.32 | 2507 | 100 |
| 17R005399 | 1580.29 | 1365.97 | 1363.74 | 44 | 96.39 | 1874.95 | 99.94 |
| 17R005400 | 918.03 | 812.86 | 811.29 | 45.28 | 95.12 | 2304.83 | 100 |
| 17R005401 | 726.5 | 649.65 | 648.45 | 47.31 | 94.3 | 3621.91 | 100 |
| 17R005402 | 1415.67 | 1264.11 | 1260.17 | 44.08 | 95.16 | 2079.9 | 99.99 |
| 17R005403 | 1164.83 | 1017.63 | 1015.8 | 44.51 | 95.64 | 1405.39 | 99.65 |
| 17R005404 | 767.75 | 673.42 | 672.03 | 45.39 | 94.69 | 1694.16 | 100 |
| 17R005405 | 1030.44 | 888.39 | 886.42 | 44.67 | 94.86 | 2277.35 | 100 |
| 17R005406 | 808.5 | 705.02 | 703.1 | 44.51 | 93.99 | 1495.34 | 99.5 |
| 17R005407 | 1131.36 | 993.17 | 991.36 | 43.14 | 94.4 | 2827.33 | 99.95 |
| 17R005408 | 1101.53 | 923.59 | 919.85 | 44.3 | 93.84 | 969.41 | 98.41 |
| 17R005409 | 1394.82 | 1171.35 | 1169.6 | 44.09 | 96.55 | 1705.64 | 100 |
| 17R005410 | 976.23 | 860.29 | 857.97 | 44.7 | 95.27 | 2255.29 | 100 |
| 17R005411 | 1543.69 | 1288.18 | 1286.54 | 43.98 | 96 | 1500.73 | 99.15 |
| 17R005412 | 1635.81 | 1470.59 | 1466.97 | 45.38 | 94.73 | 5411.33 | 100 |
| 17R005413 | 478.9 | 429.03 | 428.34 | 47.49 | 93.86 | 2388.78 | 100 |
| 17R005414 | 1124.57 | 965.94 | 964.16 | 43.87 | 95.88 | 1225.81 | 99.66 |
| 17R005415 | 615.53 | 555.72 | 554.53 | 47.29 | 93.81 | 3189.47 | 100 |
| 17R005416 | 434.04 | 389.01 | 388.29 | 48.77 | 92.42 | 2256.38 | 100 |
| 17R005417 | 723.27 | 650.64 | 649.46 | 45.42 | 94.48 | 3249.29 | 100 |
| 17R005418 | 738.98 | 630.9 | 629.06 | 43.7 | 93.75 | 1343.51 | 99.14 |
| 17R005419 | 520.34 | 470.44 | 469.33 | 46.69 | 93.86 | 2761.82 | 100 |
| 17R005420 | 545.48 | 488.18 | 487.49 | 45.92 | 95.08 | 2974.98 | 100 |
| 17R005421 | 1721.35 | 1525.03 | 1521.17 | 46.74 | 95.23 | 7336.1 | 100 |
| 17R005422 | 625.5 | 568.74 | 567.38 | 47.04 | 93.54 | 2918.3 | 100 |
| 17R005423 | 736.53 | 652.07 | 649.92 | 45.92 | 94.05 | 2433.08 | 100 |
| 17R005424 | 785.21 | 705.7 | 704.27 | 47.06 | 94.25 | 4079.4 | 100 |
| 17R005425 | 802.19 | 696.51 | 694.96 | 45.49 | 95.01 | 1827.82 | 99.56 |
| 17R005426 | 654.56 | 552.2 | 551.11 | 47.12 | 94.27 | 3195.85 | 100 |
| 17R005427 | 1121.34 | 995.35 | 991.84 | 44.23 | 94.11 | 2366.83 | 100 |
| 17R005428 | 1648 | 1452.9 | 1445.58 | 43.94 | 93.38 | 3772.79 | 100 |
| 17R005429 | 385.41 | 344.69 | 343.45 | 47.06 | 91.72 | 1763.01 | 99.93 |
| 17R005430 | 1569.79 | 1394.22 | 1390.95 | 45.36 | 95.01 | 5260.16 | 100 |
| 17R005431 | 616.89 | 554.54 | 553.53 | 46.52 | 93.58 | 2789.4 | 100 |
| 17R005432 | 1648 | 1399.71 | 1398.7 | 44.19 | 96.53 | 5896.94 | 100 |
| 17R005433 | 473.52 | 424.16 | 422.85 | 46.6 | 92.38 | 2159.48 | 100 |
| 17R005434 | 1234.45 | 1033.61 | 1031.99 | 45.02 | 95.32 | 1380.36 | 99.68 |
| 17R005435 | 859.92 | 755.62 | 754.12 | 44.94 | 95.31 | 2033.02 | 100 |
| 17R005436 | 1248.4 | 1089.68 | 1087.83 | 44.18 | 95.78 | 1365.15 | 99.53 |
| 17R005437 | 1119.85 | 969.89 | 967.75 | 44.47 | 95.48 | 1194.8 | 99.54 |
| 17R005438 | 2022.34 | 1795.2 | 1790.29 | 44.21 | 95.08 | 5810.27 | 100 |
| 17R005439 | 943.33 | 836.49 | 834.65 | 43.81 | 95.43 | 2093.63 | 99.62 |
| 17R005440 | 1701.75 | 1445.89 | 1444.2 | 43.86 | 95.86 | 1624.92 | 99.14 |
| 17R005441 | 974.81 | 837.44 | 834.81 | 43.69 | 94.92 | 1740.1 | 99.93 |
| 17R005442 | 1702.7 | 1514.4 | 1509.36 | 44.63 | 95.13 | 4297.35 | 100 |
| 17R005443 | 1211.26 | 1079.29 | 1076.87 | 46.21 | 95.56 | 3559.53 | 100 |
| 17R005444 | 1467.78 | 1270.75 | 1267.67 | 46.79 | 95.4 | 3972.55 | 100 |
| 17R005445 | 1440.44 | 1279.26 | 1276.1 | 46.7 | 95.29 | 3814.3 | 100 |
| 17R005446 | 655.8 | 586.03 | 584.68 | 46.79 | 95.49 | 2687.99 | 100 |
| 17R005447 | 825.51 | 730.42 | 728.96 | 46.14 | 95.54 | 3121.49 | 100 |
| 17R005448 | 1416.9 | 1243.7 | 1241.27 | 45.28 | 94.91 | 4169.85 | 100 |
| 17R005449 | 1141.51 | 1000.21 | 996.15 | 46.1 | 93.72 | 4572.8 | 100 |
| 17R005450 | 840.61 | 744.92 | 741.53 | 45.03 | 93.55 | 3237.45 | 100 |
| 17R005451 | 609.02 | 523.14 | 520.35 | 46.62 | 92.35 | 2519.6 | 100 |
| 17R005452 | 549.87 | 487.2 | 485.32 | 46.11 | 93.42 | 2217.03 | 100 |
| 17R005453 | 656.22 | 563 | 559.32 | 44.63 | 91.84 | 2004.23 | 100 |
| 17R005454 | 1000.42 | 860.11 | 856.84 | 45.47 | 94.67 | 4572.63 | 100 |
| 17R005455 | 538.62 | 472.16 | 470.56 | 44.86 | 94.15 | 1993.23 | 99.94 |
| 17R005456 | 1367.51 | 1220.71 | 1215.82 | 45.63 | 93.74 | 5218.47 | 100 |
| 17R005457 | 608.22 | 538.08 | 536.32 | 46.31 | 93.75 | 2487.51 | 100 |
| 17R005458 | 177.28 | 148.64 | 139.86 | 45.32 | 81.18 | 511.19 | 96.89 |
| 17R005459 | 729.69 | 643.54 | 640.9 | 45.36 | 93.62 | 2949.12 | 100 |
| 17R005460 | 378.43 | 326.39 | 324.49 | 45.35 | 92.48 | 1262.27 | 99.83 |
| 17R005461 | 842.17 | 740.76 | 736.77 | 45.37 | 93 | 3113.68 | 100 |
| 17R005462 | 681.71 | 601.54 | 599.7 | 46.47 | 94.23 | 2352.48 | 100 |
| 17R005463 | 1562.49 | 1380.46 | 1377 | 45.7 | 94.72 | 5320.15 | 100 |
| 17R005464 | 1075.43 | 972.95 | 970.81 | 45.72 | 95.2 | 4012.4 | 100 |
| 17R005465 | 1248.46 | 1056.72 | 1054.04 | 45.08 | 94.03 | 3426.48 | 100 |
| 17R005466 | 604.34 | 535.64 | 533.4 | 45.97 | 94.12 | 2120.78 | 100 |
| 17R005467 | 1841.24 | 1598.87 | 1594.29 | 47.12 | 94.53 | 7257.98 | 100 |
| 17R005468 | 798.16 | 714.05 | 711.08 | 45.66 | 93.47 | 2644.65 | 100 |
| 17R005469 | 1341.94 | 1199.51 | 1195.97 | 44.16 | 95.11 | 3844.39 | 100 |
| 17R005470 | 1280.35 | 1129.03 | 1126.66 | 46.3 | 95.46 | 5221.9 | 100 |
| 17R005471 | 972.6 | 856.32 | 854.54 | 45.95 | 95.4 | 3547.17 | 100 |
| 17R005472 | 788.98 | 714.88 | 713.26 | 46.57 | 94.16 | 3856.99 | 100 |
| 17R005473 | 1133.23 | 999.15 | 994.88 | 45 | 93.6 | 4405.96 | 100 |
| 17R005474 | 577.81 | 509.26 | 507.06 | 45.55 | 93.49 | 2090.37 | 100 |
| 17R005475 | 603.83 | 526.84 | 523.61 | 46.31 | 92.48 | 2080.41 | 100 |
| 17R005476 | 727.07 | 623.15 | 620.93 | 46.04 | 93.48 | 3144.27 | 100 |
| 17R005477 | 1118.41 | 958.13 | 954.05 | 44.54 | 93.41 | 3667.03 | 100 |
| 17R005478 | 467.91 | 412.11 | 408.86 | 46.02 | 90.92 | 1628.22 | 100 |
| 17R005479 | 809.63 | 693.67 | 690.48 | 44.93 | 93.66 | 2841.14 | 100 |
| 17R005480 | 552.57 | 484.67 | 482.88 | 46.11 | 93.53 | 2431.57 | 99.95 |
| 17R005481 | 730.14 | 626.02 | 622.44 | 45.77 | 92.56 | 2613.91 | 100 |
| 17R005482 | 849.87 | 739.69 | 734.53 | 45.43 | 90.93 | 2587.74 | 100 |
| 17R005483 | 192.65 | 169.28 | 168.24 | 47.78 | 92.08 | 884.73 | 99.46 |
| 17R005484 | 833.01 | 728.62 | 724.17 | 45.43 | 92.43 | 2700.86 | 100 |
| 17R005485 | 735.78 | 645.75 | 643.45 | 45.37 | 95.03 | 3301.03 | 100 |
| 17R005486 | 1183.54 | 1060.98 | 1056.53 | 45.35 | 93.36 | 4109.6 | 100 |
| 17R005487 | 1281.23 | 959.77 | 955.65 | 45.28 | 92.81 | 3431.4 | 100 |
| 17R005605 | 806.07 | 724.7 | 723.2 | 45.37 | 94.18 | 3602.01 | 100 |
| 17R005606 | 424.6 | 378.45 | 377.34 | 44.99 | 92.5 | 1739.36 | 99.61 |
| 17R005607 | 640.51 | 579.35 | 577.92 | 47.02 | 93.85 | 3202.24 | 100 |
| 17R005608 | 976.14 | 862.46 | 860.54 | 45.41 | 95.68 | 3617.82 | 100 |
| 17R005609 | 789.33 | 703.33 | 702.49 | 46.38 | 94.53 | 3834.03 | 100 |
| 17R005610 | 1029.81 | 924.89 | 922.01 | 46.41 | 94.52 | 3930.61 | 100 |
| 17R005670 | 794.13 | 654.26 | 651.81 | 45.82 | 93.78 | 1013.15 | 99.57 |
| 17R005690 | 172.76 | 137.31 | 137.07 | 45.94 | 95.04 | 348.16 | 97.45 |
| 17R005734 | 447.2 | 377.85 | 374.83 | 46.14 | 90.36 | 1690.2 | 99.17 |
| 17R005735 | 728.06 | 655.86 | 653.47 | 46.95 | 93.96 | 4020.29 | 100 |
| 17R005736 | 996.63 | 900.33 | 896.26 | 47.44 | 92.36 | 4928.86 | 100 |
| 17R005737 | 435.02 | 386.69 | 385.29 | 44.73 | 94.64 | 1101.64 | 100 |
| 17R005738 | 1076.29 | 951.94 | 948.02 | 46.38 | 92.68 | 4879.46 | 100 |
| 17R005739 | 833.65 | 742.83 | 735.1 | 46.31 | 89.74 | 2842.2 | 99.88 |
| 17R005740 | 549.49 | 495.65 | 494.14 | 44.92 | 94.81 | 1630.95 | 100 |
| 17R005741 | 512.86 | 463.06 | 461.5 | 44.49 | 95.12 | 1492.83 | 100 |
| 17R005742 | 1034.36 | 887.35 | 880.47 | 44.48 | 90.76 | 3385.78 | 99.93 |
| 17R005743 | 1358.76 | 1181.27 | 1175.52 | 45.05 | 92.44 | 5038.15 | 100 |
| 17R005744 | 475.47 | 424.78 | 423.46 | 44.71 | 94.92 | 1382.79 | 99.95 |
| 17R005745 | 908.94 | 809.16 | 805.47 | 45.41 | 94.1 | 2566.13 | 100 |
| 17R005746 | 419.55 | 379.93 | 378.8 | 44.99 | 95.27 | 1288.47 | 100 |
| 17R005747 | 164.17 | 136.05 | 134.59 | 49.69 | 89.49 | 760.68 | 99.27 |
| 17R005748 | 800.08 | 711.65 | 709.52 | 45.48 | 95.24 | 2421.89 | 100 |
| 17R005749 | 984.36 | 888.75 | 885.71 | 45.52 | 93.17 | 4029.46 | 100 |
| 17R005750 | 730.19 | 639.25 | 637.12 | 47.82 | 92.77 | 3884.52 | 100 |
| 17R005751 | 784.42 | 691.62 | 689.63 | 44.07 | 95.62 | 2462.24 | 100 |
| 17R005752 | 89.85 | 74.28 | 73.78 | 47.05 | 92.8 | 304.03 | 99.27 |
| 17R005753 | 969.66 | 867.76 | 862.18 | 41.53 | 93.79 | 1567.62 | 97.2 |
| 17R005754 | 1310.34 | 1143.6 | 1139.76 | 44.88 | 93.67 | 6419.37 | 100 |
| 17R005755 | 1303.65 | 1126.72 | 1119.85 | 45.05 | 91.63 | 4740.77 | 100 |
| 17R005756 | 841.21 | 745.12 | 742 | 45.26 | 92.96 | 3675.73 | 100 |
| 17R005757 | 986.65 | 865.86 | 860.85 | 42.21 | 93.84 | 1625.41 | 99.53 |
| 17R005758 | 876.79 | 765.7 | 763.52 | 43.39 | 95.87 | 2663.71 | 99.88 |
| 17R005759 | 561.2 | 484.76 | 481.77 | 43.8 | 92.6 | 1375.18 | 98.65 |
| 17R005760 | 459.56 | 400.56 | 399.46 | 45.12 | 95.1 | 1280.19 | 99.73 |
| 17R005761 | 621.82 | 544.41 | 541.07 | 45.75 | 91.15 | 2288.37 | 99.49 |
| 17R005762 | 559.32 | 485.57 | 483.86 | 43.27 | 95.19 | 1140.27 | 98.61 |
| 17R005763 | 743.97 | 667.02 | 665.08 | 44.09 | 95.49 | 2078.31 | 100 |
| 17R005764 | 806.23 | 690.63 | 688.72 | 44.41 | 95.34 | 2023.59 | 100 |
| 17R005765 | 534.52 | 453.33 | 450.27 | 43.04 | 93.08 | 1281.1 | 98.04 |
| 17R005766 | 569.71 | 486.61 | 485.06 | 44.09 | 94.28 | 1222.96 | 99.04 |
| 17R005767 | 601.71 | 542.07 | 540.74 | 45.18 | 95.61 | 1918.74 | 100 |
| 17R005768 | 861.29 | 760.45 | 754.98 | 44.86 | 91.19 | 2971.17 | 99.93 |
| 17R005769 | 627.2 | 565.88 | 563.44 | 40.85 | 94.44 | 1127.05 | 98.51 |
| 17R005770 | 691.81 | 605.72 | 602.76 | 43.84 | 94.01 | 1394.58 | 99.93 |
| 17R005771 | 1101.44 | 984.48 | 980.01 | 45.86 | 92.62 | 4611.2 | 100 |
| 17R005772 | 982.67 | 863.76 | 857.03 | 46.7 | 90.9 | 4044.26 | 100 |
| 17R005773 | 757.53 | 673.46 | 671.24 | 44.91 | 94.87 | 1804.02 | 100 |
| 17R005774 | 570.66 | 507.92 | 505.83 | 45.1 | 94.28 | 1698.64 | 99.33 |
| 17R005775 | 696.18 | 603.49 | 601.61 | 45.25 | 95.33 | 1628.14 | 99.95 |
| 17R005776 | 807.97 | 705.98 | 703.41 | 42.55 | 94.82 | 1818.96 | 99.29 |
| 17R005777 | 731.43 | 648.04 | 645.66 | 43.8 | 94.66 | 2166.4 | 99.56 |
| 17R005778 | 661.57 | 585.39 | 583.55 | 45.49 | 95.24 | 2202.79 | 100 |
| 17R005779 | 425.03 | 375.25 | 373.71 | 46.5 | 92.75 | 1698.08 | 99.35 |
| 17R005780 | 695.65 | 625.65 | 623.49 | 45.72 | 94.62 | 2431.33 | 100 |
| 17R005781 | 1009.42 | 904.37 | 900.36 | 45.77 | 92.99 | 4754.11 | 100 |
| 17R005782 | 750.58 | 664.36 | 661.08 | 47.3 | 92.08 | 3374.24 | 100 |
| 17R005783 | 892.2 | 791.56 | 789.37 | 45.41 | 95.42 | 3639.41 | 100 |
| 17R005784 | 919.62 | 808.43 | 805.82 | 44.49 | 95.05 | 3017.27 | 100 |
| 17R005785 | 485.57 | 419.84 | 418.33 | 43.72 | 94.17 | 1054.17 | 98.64 |
| 17R005786 | 887.68 | 769.93 | 766.42 | 43.94 | 94.73 | 2042.43 | 99.57 |
| 17R005787 | 919.15 | 793.47 | 791.52 | 45.54 | 95.52 | 3762.84 | 100 |
| 17R005788 | 122.12 | 103.19 | 102.43 | 45.56 | 90.65 | 463.82 | 97.18 |
| 17R005789 | 1408.58 | 1249.89 | 1244.18 | 44.07 | 93.39 | 5031.79 | 99.99 |
| 17R005790 | 762.93 | 648.76 | 645.05 | 44.06 | 93.51 | 1569.82 | 99.88 |
| 17R005791 | 449.48 | 392.85 | 390.24 | 45.42 | 91.47 | 1769.46 | 99.59 |
| 17R005792 | 812 | 705.75 | 703.37 | 45.69 | 95.04 | 2535.36 | 100 |
| 17R005793 | 1037.09 | 927.23 | 924.5 | 44.26 | 95.4 | 2927.47 | 100 |
| 17R005794 | 631.07 | 554.37 | 552.7 | 45.32 | 95.39 | 1658.01 | 100 |
| 17R005795 | 763.86 | 678.87 | 675.92 | 46.07 | 92.83 | 3529.15 | 99.74 |
| 17R005796 | 433.33 | 363.08 | 361.9 | 45.1 | 94.37 | 1026.67 | 99.94 |
| 17R005797 | 667.3 | 597.74 | 596.23 | 45.67 | 96.05 | 2801.1 | 100 |
| 17R005798 | 782.26 | 689.88 | 687.63 | 45.05 | 94.83 | 2569.99 | 100 |
| 17R005799 | 701.16 | 610.53 | 606.01 | 41.2 | 92.64 | 1304.57 | 96.89 |
| 17R005800 | 1016.81 | 894.8 | 892.14 | 44.81 | 95.45 | 3042.01 | 100 |
| 17R005801 | 1077.89 | 959.8 | 954.86 | 46.33 | 92.49 | 5022.58 | 100 |
| 17R005802 | 688.83 | 589.87 | 587.96 | 44 | 94.5 | 2053.67 | 100 |
| 17R005803 | 664.17 | 589.12 | 587.17 | 43.86 | 95.59 | 1801.4 | 99.94 |
| 17R005804 | 1043.74 | 929.4 | 925.4 | 43.53 | 95.14 | 2675.22 | 100 |
| 17R005805 | 980.62 | 835.25 | 832.37 | 44.14 | 94.77 | 3018.76 | 99.68 |
| 17R005806 | 464.94 | 408.61 | 405.99 | 43.85 | 92.98 | 916.19 | 99.27 |
| 17R005807 | 1007.83 | 883.93 | 881.43 | 44.23 | 95.86 | 3341.01 | 100 |
| 17R005808 | 599.53 | 515.85 | 514.07 | 44.04 | 94.65 | 1720.91 | 98.89 |
| 17R005809 | 916.4 | 818.75 | 816.5 | 44.81 | 96.01 | 3186.27 | 100 |
| 17R005810 | 969.94 | 878 | 873.11 | 47 | 91.83 | 4658.79 | 100 |
| 17R005811 | 450.09 | 388.02 | 386.88 | 44.85 | 95.23 | 1076.28 | 100 |
| 17R005812 | 691.28 | 619.53 | 617.04 | 44.68 | 94.74 | 1838.78 | 100 |
| 17R005813 | 741.53 | 644.87 | 640.3 | 45.22 | 90.69 | 2466.48 | 99.73 |
| 17R005814 | 704.9 | 610.49 | 603.88 | 42.63 | 89.9 | 2013.87 | 97.5 |
| 17R005815 | 693.58 | 624.86 | 622.79 | 44.71 | 95.06 | 2108.37 | 100 |
| 17R005816 | 904.95 | 819.19 | 815.93 | 45.49 | 94.22 | 3319.62 | 100 |
| 17R005817 | 326.57 | 273.15 | 271.5 | 47.09 | 90.85 | 1326.58 | 99.52 |
| 17R005818 | 497.27 | 440.51 | 438.83 | 47.17 | 93.08 | 2647.97 | 100 |
| 17R005819 | 1191.15 | 1035.69 | 1031.57 | 46.6 | 92.49 | 4911.43 | 100 |
| 17R005820 | 814.45 | 723.23 | 720.1 | 47.43 | 93.01 | 4252.46 | 100 |
| 17R005821 | 883.95 | 800.13 | 797.3 | 44.04 | 95.2 | 2345.44 | 100 |
| 17R005825 | 768.12 | 665.52 | 662.24 | 45.56 | 92.69 | 3030.16 | 99.78 |
| 17R005826 | 851 | 758.39 | 754.45 | 47.13 | 91.84 | 4079.32 | 100 |
| 17R005827 | 719.25 | 644.97 | 642.26 | 45.41 | 92.85 | 3285.39 | 99.87 |
| 17R005828 | 851.85 | 747.42 | 744.18 | 45.47 | 94.07 | 2987.4 | 100 |
| 17R005829 | 852.84 | 774.72 | 770.52 | 47.13 | 91.74 | 4343.49 | 100 |
| 17R005830 | 390.18 | 347.51 | 346.19 | 45.61 | 94.13 | 894.97 | 99.91 |
| 17R005831 | 660.28 | 553.26 | 548.85 | 41.05 | 92.07 | 1026.14 | 96.99 |
| 17R005832 | 442.1 | 392.82 | 390.55 | 47.27 | 91.75 | 1952.2 | 99.16 |
| 17R005833 | 847.24 | 743.01 | 737.85 | 46.47 | 91.39 | 3445.24 | 99.62 |
| 17R005834 | 1068.66 | 952.1 | 947.65 | 45.1 | 93.12 | 4279.11 | 100 |
| 17R005835 | 555.04 | 481.42 | 479.17 | 44.87 | 93.44 | 1617.71 | 98.42 |
| 17R005836 | 664.66 | 579.73 | 577.49 | 44.88 | 94 | 1981.77 | 99.16 |
| 17R005837 | 1060.35 | 947.97 | 944.53 | 46.5 | 93.68 | 5876.34 | 100 |
| 17R005838 | 857.64 | 773.25 | 770.02 | 45.14 | 94.23 | 3056.39 | 99.63 |
| 17R005839 | 924.46 | 795.11 | 792.41 | 45.33 | 92.92 | 3879.48 | 99.68 |
| 17R005840 | 730.34 | 649.05 | 647.53 | 45.87 | 95.58 | 2503.33 | 100 |
| 17R005841 | 789.1 | 700.74 | 697.46 | 45.83 | 92.41 | 3118.37 | 99.94 |
| 17R005842 | 908.25 | 789.25 | 786.97 | 46.17 | 94.27 | 4467.85 | 100 |
| 17R005843 | 383.89 | 334.43 | 332.66 | 44.33 | 94.83 | 809.06 | 99.39 |
| 17R005844 | 759.23 | 672.3 | 670.1 | 43.95 | 95.6 | 1986.03 | 100 |
| 17R005845 | 958.96 | 855.12 | 850.69 | 45.67 | 92.6 | 3819.98 | 99.99 |
| 17R005846 | 850.81 | 765.43 | 761.74 | 46.18 | 92.78 | 3730.19 | 100 |
| 17R005847 | 1243.93 | 1086.06 | 1080.21 | 43.96 | 92.26 | 4540.39 | 100 |
| 17R005848 | 825.08 | 726.56 | 722.61 | 45.14 | 94.31 | 2094.25 | 100 |
| 17R005849 | 716.48 | 606.11 | 602.34 | 41.66 | 93.05 | 1351.72 | 98.39 |
| 17R005850 | 1107.93 | 988.3 | 982.67 | 45.82 | 92.1 | 4218.15 | 100 |
| 17R005851 | 383.05 | 344.5 | 343.37 | 44.38 | 96.01 | 1064.98 | 99.43 |
| 17R007415 | 694.25 | 589.01 | 587.88 | 45.06 | 95.47 | 1483.1 | 100 |
| 17R007416 | 252.05 | 223.14 | 221.64 | 40.72 | 95.62 | 494.27 | 96.3 |
| 17R007424 | 548.75 | 463.3 | 462.79 | 42.91 | 96.54 | 1165.87 | 99.16 |
| 17R007427 | 497.54 | 420.51 | 419.59 | 43.36 | 95.69 | 966.98 | 99.28 |
| 17R007429 | 340.67 | 281.95 | 281.58 | 42.96 | 95.9 | 633.54 | 97.31 |
| 17R007430 | 514.07 | 433.36 | 432.67 | 44.61 | 95.62 | 1092.16 | 99.61 |
| 17R005914 | 1044.57 | 927.16 | 924.3 | 41.56 | 95.98 | 2375.45 | 99 |
| 17R005917 | 826.43 | 713.14 | 710.69 | 43.62 | 95.47 | 2123.31 | 100 |
| 17R005919 | 1552.51 | 1286.79 | 1282.42 | 42.81 | 95.49 | 3413.23 | 100 |
| 17R007414 | 1082.16 | 935.17 | 926.76 | 39.93 | 94.81 | 1693.86 | 97.82 |
| 17R007417 | 749.76 | 658.32 | 651.92 | 39.8 | 94.47 | 1243.2 | 98.23 |
| 17R007418 | 685.56 | 595.83 | 589.36 | 39.97 | 93.66 | 983.99 | 96.89 |
| 17R007419 | 941.62 | 837.47 | 829.27 | 39.8 | 94.67 | 1473.53 | 99.82 |
| 17R007420 | 1213.57 | 1082.79 | 1073.46 | 39.85 | 94.87 | 1997.85 | 96.89 |
| 17R007421 | 999.33 | 906.86 | 898.15 | 40.16 | 94.73 | 1923.2 | 97.86 |
| 17R007422 | 1287.75 | 1161.61 | 1150.57 | 39.7 | 95.46 | 2554.07 | 98.72 |
| 17R007423 | 1095.07 | 940.41 | 932.22 | 39.7 | 94.92 | 1673.74 | 96.89 |
| 17R007425 | 1206.32 | 1072.82 | 1065.37 | 40.56 | 95.14 | 2260.21 | 99.29 |
| 17R007426 | 523.79 | 437.26 | 430.56 | 39.67 | 93.02 | 626.75 | 96.06 |
| 17R007428 | 824.73 | 733.22 | 724.87 | 40.2 | 93.56 | 1179.22 | 97.23 |
| 17R007431 | 556.79 | 462.61 | 457.07 | 39.68 | 93.58 | 751.14 | 96.74 |
| 17R007432 | 389.47 | 335.61 | 330.33 | 39.96 | 92.6 | 502.23 | 96.66 |
| 17R007433 | 1109.26 | 978.75 | 970.83 | 40.06 | 95.56 | 2321.5 | 98.44 |
| 17R007434 | 650.05 | 560.93 | 553.54 | 40.03 | 93.53 | 856.41 | 96.74 |
| 17R008866 | 661.14 | 565.71 | 563.71 | 48.4 | 92.75 | 2130.76 | 100 |

**Table S3. Comparison of mutant ratio in 15 HSCR patients carrying 13 different kinds of *RET* variants examined by targeted next-generation sequencing (NGS) and droplet digital polymerase chain reaction (ddPCR).**

| **No.** | **Variant**  **(protein change) ^a^** | **Sample ID** | **Method ^#^** | | | | | |
| --- | --- | --- | --- | --- | --- | --- | --- | --- |
|  |  |  | **NGS** | | | **ddPCR** | | |
|  |  |  | **Reference-**  **allele reads** | **Alternative-allele reads** | **Mutant ratio (%)** | **Reference-allele reads** | **Alternative-allele reads** | **Mutant ratio (%)** |
| 1 | p.(Ser32Leu) | HSCRFM197 | 285 | 194 | 40.5 | 1482 | 1388 | 48.4 |
|  |  | HSCRFM198 | 772 | 564 | 42.2 | 1830 | 1836 | 50.1 |
| 2 | p.(Gly93Ser) | XHYY057 | 497 | 436 | 46.7 | 2044 | 1994 | 49.4 |
| 3 | p.(Arg180*) | HSCRFM191 | 1171 | 1110 | 48.7 | 1840 | 2000 | 52.1 |
| 4 | p.(Arg231Cys) | XHYY022 | 637 | 650 | 50.5 | 1594 | 1582 | 49.8 |
| 5 | p.(Val282Valfs*71) | XHYY051 | 442 | 463 | 51.2 | 1836 | 1824 | 49.8 |
| 6 | p.(Gln421Pro) | XHYY087 | 608 | 610 | 50.1 | 1938 | 1956 | 50.2 |
| 7 | p.(Gly605Asp) | HSCRFM181 | 446 | 441 | 49.7 | 112 | 108 | 49.1 |
| 8 | p.(Gly731Glu) | XHYY093 | 683 | 705 | 50.8 | 1750 | 1692 | 49.2 |
| 9 | p.(Arg770*) | HSCRFM072 | 350 | 307 | 46.7 | 1524 | 1654 | 52.1 |
| 10 | p.(Arg897Gln) | HSCRFM230 | 114 | 129 | 53.1 | 2380 | 2376 | 50.0 |
|  |  | HSCRFM075 | 427 | 379 | 47.0 | 2160 | 2342 | 52.0 |
| 11 | p.(Tyr1062Cys) | HSCRFM024 | 1131 | 1149 | 50.4 | 1712 | 1682 | 49.6 |
| 12 | p.(Arg1089Arg) | HSCRFM007 | 728 | 688 | 48.6 | 2160 | 2102 | 49.3 |
| 13 | c.2608-3C>G | HSCRFM156 | 211 | 162 | 43.4 | 2128 | 2072 | 49.3 |
| **Total** | **Average** |  |  |  | **48.0** |  |  | **50.0** |
|  | **Standard deviation** |  |  |  | **3.6** |  |  | **1.1** |

^a^ RefSeq NM_020975.5, NP_066124.1.

^#^ Variants covered less than 500X are highlighted in blue.
